# Impacts of Cetylpyridinium Chloride on the Survival, Development, Behavior, and Oxidative Stress of Early-Life-Stage Zebrafish (*Danio rerio*)

**DOI:** 10.3390/antiox11040676

**Published:** 2022-03-30

**Authors:** Xuchun Qiu, Michaela Sia Tengbe, Xingyi Xia, Kejun Dong, Chen Chen, Yanhong Shi, Ming Li, Hai Xu, Xiangyang Wu, Kun Chen

**Affiliations:** 1 Institute of Environmental Health and Ecological Security, School of the Environment and Safety Engineering, Jiangsu University, Zhenjiang 212013, China; xuchunqiu@ujs.edu.cn (X.Q.); mtengbe@gmail.com (M.S.T.); xxy18487431569@163.com (X.X.); dkjcl0516@163.com (K.D.); chenchen9688@yeah.net (C.C.); shiyanhong2003@126.com (Y.S.); liming@ujs.edu.cn (M.L.); xuhai@ujs.edu.cn (H.X.); wuxy@ujs.edu.cn (X.W.); 2 Jiangsu Collaborative Innovation Center of Technology and Material of Water Treatment, Suzhou University of Science and Technology, Suzhou 215009, China

**Keywords:** behavioral responses, cetylpyridinium chloride, development, early life stages, oxidative stress, zebrafish

## Abstract

Cetylpyridinium chloride (CPC) is a widely used surfactant that has been detected in various water ecosystems. However, knowledge on the toxicity of CPC to fish remains scarce. Here, we examined the survival, development, behavior, and oxidative stress in the early life stages of zebrafish exposed to CPC (0, 4, 40, 400, and 1200 μg/L) until 120 h post-fertilization (hpf). Results showed that CPC induced significant mortality at 400 and 1200 μg/L, with a 120 h-EC_50_ value of 175.9 μg/L. CPC significantly decreased the heart rate of embryos (48 hpf; 4–400 μg/L) and larvae (72 hpf; 40 and 400 μg/L). At 120 hpf, CPC exhibited a dual effect on the locomotion activity (decreased at 400 μg/L and increased at 4 and 40 μg/L) and elevated the reactive oxygen species, superoxide dismutase, and glutathione levels in zebrafish larvae at 400 µg/L. In addition, a correlation analysis revealed that CPC-induced oxidative stress might play a critical role in mediating the cardiac and behavioral toxicity of CPC to zebrafish larvae. Our findings suggest that CPC may disturb the fish’s development, behavior, and oxidative status at environmentally relevant concentrations, which should not be ignored when assessing its potential risks to aquatic ecosystems.

## 1. Introduction

The global annual production and consumption of quaternary ammonium salts (QAS) keep increasing to meet the demand of the ever-increasing human population [1,2,3,4]. For example, the surfactant cetylpyridinium chloride (CPC, a typical QAS) has been widely used as the active ingredient (0.01–1% *w/w*) or detergent additive (up to 5 mg/L) in personal care products [2,3]. Recently, CPC has been accepted for use in food processing to fight against microbial contamination [4]. After their use, however, products containing CPC either end up in wastewater treatment plants (WWTPs) or are directly discharged into the natural environment [5]. Although more than half of CPC could be removed from waste streams through biodegradation and adsorption [6], CPC residues can still be detected at levels of up to 52 μg/L in some river water and groundwater samples [7,8]. Moreover, the concentrations of CPC detected in surface water samples have exceeded its 50% lethal concentration (LC_50_) in freshwater planaria [9] and some amphibians [10]. Thus, the potential risk of CPC to aquatic species has sparked considerable interest [10,11,12].

It has been reported that surfactants might affect the survival, growth, development, and swimming behaviors of aquatic species [10,11,12,13]. Generally, the toxicity of a surfactant relates to its ability to absorb and infiltrate the cell membrane of aquatic species [14]. Regarding CPC, Park et al. [10] reported that embryonic exposure to CPC (540 and 720 µg/L for 7 days) significantly affected the survival, development, and growth of *Bombina orientalis*. Recently, Bhattacharya and colleagues reported that CPC could detrimentally modulate the levels of oxidative stress enzymes in the worms of *Branchiura sowerbyi* (exposed at levels of 12 and 36 µg/L for 1, 7, and 14 days) and *Tubifex tubifex* (exposed at levels of 21.3 and 61.9 µg/L for 14 days) [15,16] and adversely affect the antioxidant enzymes in the liver of *Cyprinus carpio* (exposed at levels of 2 and 6 µg/L for 15, 30, and 45 days) [17]. Moreover, Mustapha and Bawa-Allah [13] also found that both anionic and nonionic surfactants could affect the antioxidant enzymes and the liver function of African sharp tooth catfish *(Clarias gariepinus*).

Oxidative stress results from an imbalance between reactive oxygen species (ROS) production and antioxidant defenses in living organisms [18]. Elevated ROS can cause the oxidation of proteins and lipids, alterations in gene expression, and changes in cellular redox status [19]. Correspondingly, organisms have evolved antioxidant defense systems—e.g., superoxide dismutase (SOD), catalase (CAT), glutathione s-transferase (GST), glutathione (GSH), and glutathione-peroxidase (GPX)—to protect cells from attack by ROS [18]. As a product of oxidative stress-mediated lipid peroxidation, malondialdehyde (MDA) is often used to evaluate the oxidative damage status in organisms [20]. In addition, MDA also acts as a mitochondrial toxin that may cause significant mitochondrial dysfunction by the inhibition of respiration and the inactivation of important enzymes [21]. Furthermore, oxidative stress induced by environmental pollutants is also of ecological significance, particularly in the aquatic environment, which provides a sink for many pollutants [22]. For example, oxidative stress induced by pollutants can trigger behavioral impairments in teleosts, subsequently decreasing their fitness and survival in natural ecosystems [22,23]. However, knowledge on the toxicity of CPC to fish and its mechanistic links to the oxidative stress induced remain scarce.

Zebrafish (*Danio rerio*) is a model organism used for assessing the ecotoxicological effects of chemicals [24,25,26,27]. As an alternative to the acute fish toxicity test, the zebrafish embryo test has been optimized, standardized, and validated by the OECD [28] and has been wildly used to investigate the links between chemical-induced oxidative stress and its toxic effects [29,30,31,32,33]. In this study, therefore, we exposed zebrafish embryos to a range of concentrations of CPC and examined their survival, development, and behavioral responses within early life stages (until 120 h post-fertilization (hpf)). Then, variations in the levels of ROS, SOD, GSH, and CAT were examined at 120 hpf. This study aimed to investigate the effects of CPC on the survival, development, behavior, and oxidative stress in the early life stages of zebrafish and explore their possible mechanistic links.

## 2. Materials and Methods

### 2.1. Chemical

Cetylpyridinium chloride (123-03-5, C_21_H_38_ClN) was purchased from the Shanghai Yien Chemical Technology Co., Ltd. (Shanghai, China). Other reagents (analytical grade) were purchased from the Sinopharm Chemical Reagent Co., Ltd. (Shanghai, China). The enzyme-linked immunosorbent assay (ELISA) kits used for assaying the ROS, SOD, GSH, and CAT were purchased from Bomei Biotechnology Co., Ltd. (Hefei, Anhui, China).

### 2.2. Test Organisms

Zebrafish (*Danio rerio*, AB strain) brood-stock, which was maintained in our laboratory for more than 3 months, was used to produce the embryos. The female and male zebrafish were separately cultured in two 16 L circular glass aquariums containing 12 L of dechlorinated tap water (conductivity at 0.50–0.53 mS/cm) at 27 ± 1 °C. The aquariums were kept under a 14L:10D h light:dark cycle and half of the water was renewed every two days. Zebrafish were fed with *Artemia nauplii* (<24 h after hatching, twice a day).

A total of 50 zebrafish (30 females and 20 males) were used for producing embryos. On the afternoon before the exposure test, three females and two males were gently placed in a spawning box (Aqua Schwarz GmbH, Göttingen, Germany) and separated by a baffle. While the light onset on the following morning, the baffle was removed for the spawning and fertilization. After 30 min, all embryos from the ten spawning boxes were transferred to a Petri dish containing E3 medium (5 mM NaCl, 0.17 mM KCl, 0.33 mM CaCl_2_, 0.33 mM MgSO_4_ in dH_2_O) [33], and washed three times using the E3 medium. Subsequently, healthy embryos were selected and transferred to clean Petri dishes for an exposure test.

### 2.3. Exposure Experiment

The test solutions were prepared by pipetting calculated amounts of the CPC stock solution (60 mg/mL in deionized water) into an E3 medium to obtain final concentrations of 0 (control), 4, 40, 400, and 1200 µg/L, which are equivalent to 0, 0.01, 0.1, 1, and 3 µM, respectively. For each treatment, 120 healthy embryos were randomly selected and introduced to three Petri dishes (40 healthy embryos per Petri dish, *n* = 3) containing 20 mL of the test solution. Embryos in Petri dishes were maintained in an incubator at 28 °C with the same photoperiod as the adults. The experiment was conducted for 120 h post-fertilization (hpf), and the test solutions were renewed every 24 h. The survival of embryos/larvae was confirmed 6 times daily, and dead individuals were removed immediately after the confirmation. The mortality rate was calculated at 24, 48, 72, 96, and 120 hpf. At 48 and 72 hpf, the heart rate (heartbeats per minute) of embryos (eight random individuals per Petri dish) was assessed. For this assessment, videos of embryo/larvae were recorded using a stereomicroscope equipped with a DP73 camera (SZX16, Olympus, Tokyo, Japan). Subsequently, the videos were played back at 1/2 speed and the number of heartbeats within 1 min was manually determined using a mechanical counter. In addition, the hatching time of each successfully hatched larvae (i.e., hatched within 120 hpf) was recorded for assessing the time to hatching.

### 2.4. Light/Dark Locomotion Test of Newly Hatched Larvae

At 120 hpf, zebrafish larvae (20 individuals for each Petri dish) were carefully transferred to 96-well plates, with a single larva in each well (inner diameter = 8 mm) for a light–dark locomotion test. After a 10 min acclimation period in the dark, the behavior response of larvae to the alternating cycle of light/dark shifts (i.e., dark–light–dark–light–dark; 10 min each) was examined. The locomotion of larvae was tracked by a DanioVision system (Noldus, Wageningen, The Netherlands), and the average swimming velocity (ASV, mm/s) was analyzed using the EthoVision XT software (Vision 11.5; Noldus). In addition, the ratio of the AVS in the following photoperiod to its previous photoperiod was calculated to represent the response of zebrafish larvae to light/dark shifts.

### 2.5. Biochemical Assays

At 120 hpf, ROS, SOD, GSH, CAT, and malondialdehyde (MDA) levels in zebrafish larvae were determined to evaluate the oxidative stress induced by CPC exposure. For those assays, 30 individuals were pooled as one sample and 3 replicates were used for each treatment group. The homogenate and supernatant for those assays were prepared following the method described by Shi et al. [34]. Briefly, each sample was weighed and homogenized with 9 vol (*w/v*) of phosphate buffered saline (10 mM, pH 7.2–7.4). Then, the homogenate was centrifuged at 6000× *g* for 5 min, and the supernatant was collected. The biochemical assays were conducted using the corresponding ELISA Kit (Bomei Biotechnology Co., Ltd.), for which we used the Sandwich-ELISA method. The assay procedure was conducted following the corresponding manufacturer’s instructions, and the optical density (OD) was measured using a Microplate Spectrophotometer (Synergy H4, BioTek, Winooski, VT, USA) at a wavelength of 450 nm. The concentrations in samples were calculated by comparing the OD of the samples to the standard curve. The units of oxidative parameters were normalized by the total protein amount and were expressed as per mg- of protein (/mg-P).

### 2.6. Statistical Analysis

The survival and hatching data were analyzed using an accelerated failure time (AFT) model using the eha and survival packages in R version 3.4.4, as described in our previous studies [35,36]. A generalized linear model (GzLM) was used to analyze the statistical significance in the behavioral and biochemical parameters over different treatments. A Spearman’s correlation analysis was used to test the statistical significance of the relationship between oxidative stress biomarkers and biological and behavioral parameters. The above statistical analyses were performed using SPSS 16.0 (SPSS Inc., Chicago, IL, USA).

## 3. Results

### 3.1. Effects on Embryonic Mortality and Hatching

**Figure 1:**
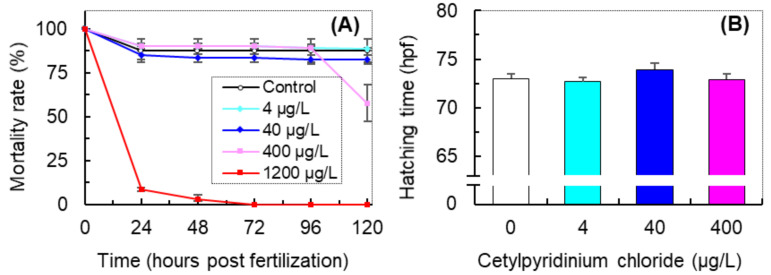
The survival curve (**A**) and average hatching time (**B**) of zebrafish (*Danio rerio*) exposed to cetylpyridinium chloride (CPC). Data are presented as means ± SE.

The effect of CPC on the survival and average hatching time is shown in Figure 1. Although exposure to CPC at 4.0 and 40 μg/L did not notably affect the survival of zebrafish, at 400 μg/L it induced a relatively higher mortality (Figure 1A). Furthermore, all the embryos exposed to CPC at a level of 1200 μg/L died within 72 hpf. An AFT model fitted those time-to-death data, and the estimated coefficients are listed in Table 1. The negative value of *d_CPC_* (−2.03 × 10^−3^, *p* < 0.01, Table 1) indicated that the CPC concentration exhibited significantly negative associations with embryo survival. The 120 h-LC_50_ of CPC in the early life stages of zebrafish was calculated to be 175.9 (60.6–611.0) μg/L by probit analysis.

As shown in Figure 1B, no significant differences in the average hatching time of zebrafish embryos were observed. The coefficients of the AFT model fitting for the time-to-hatching data also indicate that exposure to CPC at a level of 4–400 μg/L did not significantly affect the hatching of zebrafish embryos (Table 2).

### 3.2. Effects on the Heart Rate

**Figure 2:**
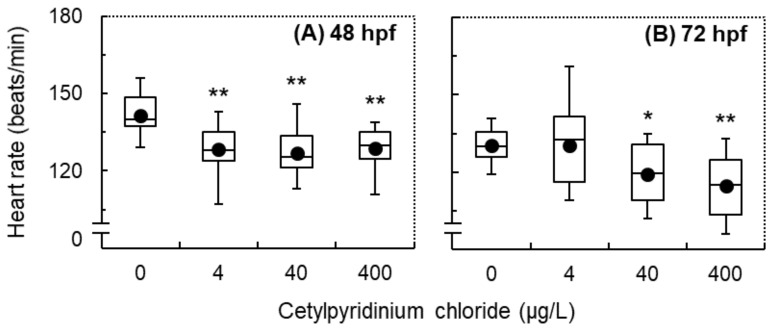
The heart rate of zebrafish (Danio rerio) exposed to cetylpyridinium chloride (CPC) at levels of 0 (control), 4.0, 40, and 400 µg/L for 48 (**A**) and 72 (**B**) hours post-fertilization (hpf). Data are presented as means ± SE (n = 18). Asterisks denote statistically significant differences between the exposure and control groups (* *p* < 0.05; ** *p* < 0.01).

In the control group, the average heart rates of zebrafish embryos at 48 and 72 hpf were 140 ± 4 and 130 ± 3 beats/min, respectively (Figure 2). At 48 hpf, significantly lower heart rates were observed in zebrafish embryos exposed to CPC at levels of 4, 40, and 400 μg/L, compared with the control (*p* < 0.01, Figure 2A). At 72 hpf, significantly lower heart rates were observed in the newly hatched larvae exposed to CPC at levels of 40 (*p* = 0.023) and 400 (*p* < 0.01) μg/L compared with the control (Figure 2B).

### 3.3. Effects on the Behavioral Traits of Zebrafish Larvae during Light/Dark Shift

**Figure 3:**
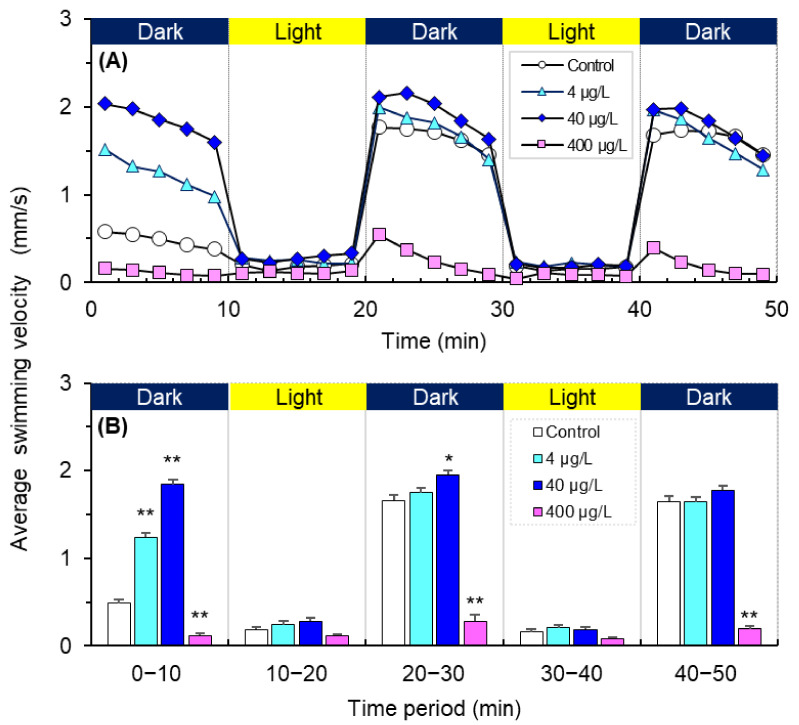
The average swimming velocity of zebrafish (*Danio rerio*) larvae exposed to cetylpyridinium chloride at levels of 0 (control), 4.0, 40, and 400 µg/L for 120 h post-fertilization. The blue and yellow bars at the top indicate the dark and illumination conditions, respectively. (**A**) Data are presented as the mean value in each 2 min interval. (**B**) Data are presented as means ± SE (*n* = 60) within each 10 min period, and asterisks denote statistically significant differences between the exposure and control groups (* *p* < 0.05; ** *p* < 0.01).

The newly hatched zebrafish larvae exhibited a similar movement pattern under this light/dark shift, i.e., a highly active state in the dark period and a resting state in the illumination period (Figure 3A). However, CPC exposure exhibited dual effects on the average swimming velocity (ASV) of zebrafish larvae (i.e., hypoactivity at 400 μg/L and hyperactivity at 4 and 40 μg/L), especially within the first 10 min dark period (*p* < 0.01, Figure 3A,B). The ASV of larvae within each 10 min period is shown in Figure 3B. In each dark period, larvae in the 40 μg/L CPC group exhibited the highest ASV, while those in the 400 μg/L CPC group exhibited the lowest ASV (Figure 3B). In light periods, the ASV of larva in the 400 μg/L CPC group was also significantly lower than that in the 40 μg/L CPC group (*p* < 0.05 for both light periods), and significantly lower than that in the 4 μg/L CPC group in the second light period (*p* < 0.05).

### 3.4. Responses in the Oxidative Stress Biomarkers and Their Relationship to Biological and Behavioral Parameters

**Figure 4:**
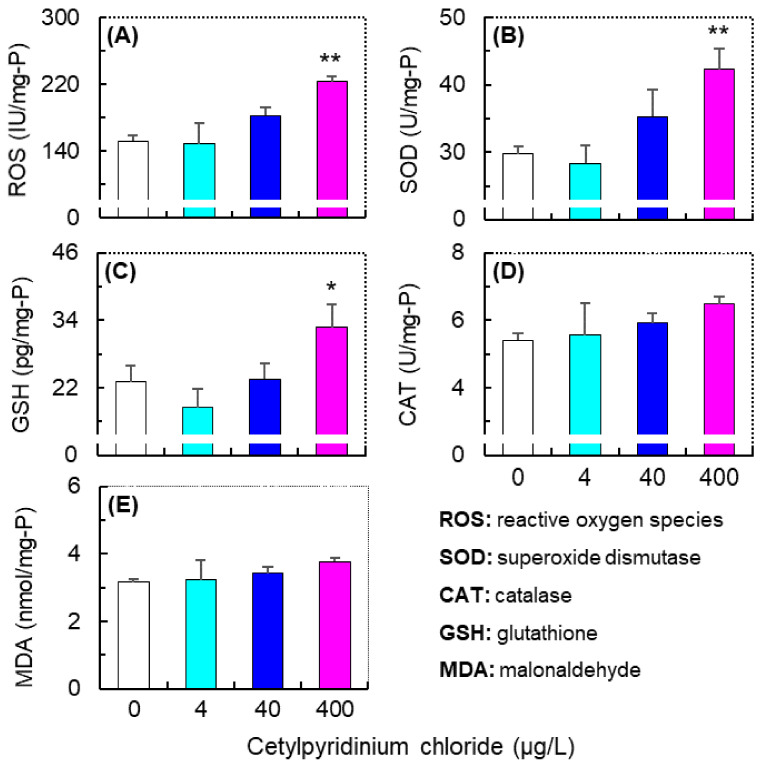
Variations in the levels of ROS (**A**), SOD (**B**), GSH (**C**), CAT (**D**) and MDA (**E**) in zebrafish larvae exposed to cetylpyridinium chloride at levels of 0 (control), 4.0, 40, and 400 µg/L for 120 h post-fertilization (hpf). Data are shown as mean ± SE (*n* = 3) and asterisks denote statistically significant differences between the exposure and control groups (* *p* < 0.05; ** *p* < 0.01).

Exposure to CPC could significantly induce oxidative stress and affect the antioxidant capacity of zebrafish larvae, especially at a level of 400 µg/L. As shown in Figure 4A, the ROS level of zebrafish larvae in the 400 µg/L CPC group was significantly higher than that in the other groups. Correspondingly, both the SOD activity and GSH concentration of zebrafish larvae in the 400 µg/L CPC group were significantly higher than those in the control (*p* < 0.01) and 4 µg/L CPC (*p* < 0.05) groups (Figure 4B,C). However, although the CAT activity and MDA level tended to increase with elevated CPC exposure concentrations, no significant difference was detected between all the experimental groups (Figure 4D,E).

The correlation between oxidative stress biomarkers and biological and behavioral parameters is shown in Table 3. Spearman’s correlation analysis shows that all the oxidative stress biomarkers exhibited a significantly negative correlation with the heart rate of zebrafish larvae (i.e., at 72 hpf, *p* < 0.01), but not with that of embryos (i.e., 48 hpf). There were no significant correlations between the oxidative stress biomarkers and the ASV within each 10 min period (Table 3). However, the levels of ROS, SOD, CAT, and MDA exhibited a significantly negative correlation with the responses to the first (i.e., D2/L1, Table 3) and second (i.e., D3/L2, Table 3) light to dark shift, and significantly positive correlations with the response to the second dark to light shift (i.e., L2/D2, Table 3). The GSH level also exhibited significantly positive correlations with the behavioral response to the second dark to light shift (i.e., L2/D2, *p* < 0.05, Table 3).

## 4. Discussion

Our results demonstrate that CPC at relatively higher concentrations (i.e., 400 and 1200 µg/L) could significantly reduce the survival of zebrafish embryos and larvae. Interestingly, a recent study [37] showed that short-term exposure to CPC (0.1 μM, 1 h) reduced the mortality and viral load of zebrafish larvae following influenza infection, which suggested a protective effect of CPC. However, the authors also found that CPC doses significantly higher than 0.1 μM (i.e., 40 μg/L) caused the mortality of zebrafish embryos [37], which is very consistent with our results. Based on the survival curve, the 120 h-LC_50_ of CPC for the early life stages of zebrafish was calculated to be 175.9 μg/L. Earlier studies have reported that the 48-h LC_50_ of CPC in freshwater planarian (*Dugesia japonica*) was 40 μg/L [9], and its 168-h LC_50_ in *B. orientalis* (Amphibia: Anura) embryos was 69.7 μg/L [10]. Considering that the CPC could be detected at up to 52 μg/L in some field water samples [7,8], this pollutant may adversely affect certain sensitive aquatic organisms [9,10]. Because the heart is the first functional organ in zebrafish development, heart rate measurement is an evident toxicological endpoint in fish embryo toxicity tests [28]. This study observed significantly decreased heart rates in zebrafish embryos (48 hpf) and larvae (72 hpf) exposed to CPC at levels of 4, 40, and 400 μg/L. Previous studies have also reported reduced heartbeats in zebrafish embryos exposed to the surfactant of stearamidopropyl dimethylamine [38] and fatty alcohol polyoxyethylene ether-7 [39]. Generally, the reduced heartbeat is related to the underdeveloped heart and pericardium [40] or the high level of apoptotic cells in the heart of fish [41,42]. Therefore, CPC exposure could be developmentally toxic and might affect the cardiac function of early-life-stage zebrafish, as evidenced by the reduction in heartbeats.

The heart rate of larvae (i.e., at 72 hpf) exhibited significant correlations with all the oxidative stress biomarkers (i.e., ROS, SOD, GSH, and CAT). Similarly, many previous studies have also reported that environmental pollutants could cause abnormal development in various organs of zebrafish by inducing oxidative stress [29,30,31,32,33]. On the other hand, ROS can also act as signal molecules to mediate embryonic development processes [43], and high ROS levels may lead to heart looping disorder during the heart development of zebrafish [44,45]. Therefore, we infer that the oxidative stress induced by CPC may play an important role in its cardiac toxicity to zebrafish larvae.

The light/dark locomotion test is a useful method to reflect the integrated function of the brain, nervous system, and visual pathways of fish larvae [46,47]. In this study, CPC exhibited dual effects on the ASV of newly hatched zebrafish larvae (i.e., hypoactivity at a level of 400 μg/L and hyperactivity at levels of 4 and 40 μg/L), especially within the first 10 min dark period. The concentration-specific impacts on fish locomotion activity have been reported in many neuroactive chemicals, such as diazepam [26], organophosphorus [48], heavy metals [49], and so on. Hyperactivity is associated with neural circuity components of the early zebrafish locomotor network [50]. Generally, hyperactivity in fish may attract a predator’s attention and reduce the energy available for essential activities [26,51]. On the other hand, hypoactivity may increase the risk of being killed and decrease predation efficiency [52,53]. Thus, these abnormal behaviors of zebrafish larvae in the exposure groups suggest that CPC may cause potential ecological risks, even at environmentally relevant concentrations.

The CPC exposure increased the ROS, SOD, and GSH levels in zebrafish larvae, especially at 400 µg/L. The accumulation of ROS in zebrafish referred to increased CPC-induced oxidative stress, which may also activate the antioxidant systems (i.e., increase the SOD and GSH activity) to combat this stress [18,54]. Previous studies have reported that CPC at levels of 21.3 and 63.9 μg/L (for 1 and 7 days) could induce oxidative stress and significantly elevate the SOD activity and GSH concentration in the sludge worm (*T. tubifex*) [16], and that at 6 μg/L (for 15, 30, and 45 days) it could significantly elevate the SOD and CAT activity in the liver of European carp (*C. carpio*) [17]. Thus, oxidative stress induced by CPC seems to be a common pathway to cause toxicity to various aquatic species. Moreover, our results also showed that the MDA level did not significantly increase, suggesting that the elevated SOD and GSH levels induced by CPC exposure may play critical roles in protecting cells from severe oxidative damage. Nevertheless, the significant correlations between the levels of biomarkers and the behavioral responses to light/dark shifts suggest that CPC-induced oxidative stress may be an important mechanism mediating the behavioral toxicity of this chemical to zebrafish larvae. In the light/dark preference test, the responses to light/dark shift have been hypothesized to be related to predator avoidance [55], light searching for foraging [56], and anxiety-related behaviors [57]. Alterations in these behaviors may affect population and have community consequences for fish species [55,56,57,58]. Therefore, our findings support the notion that oxidative stress induced by pollutants could trigger behavioral impairments and may finally result in the decreased fitness and survival of teleosts [22,23].

## 5. Conclusions

Our results demonstrate that CPC may disturb the development and behavioral response to light stimulation in the early life stages of zebrafish, even at environmentally relevant concentrations (i.e., 4 and 40 µg/L). Thus, the ecological consequences of these sublethal effects should not be ignored when assessing their potential risks to aquatic ecosystems. Moreover, our correlation analysis suggests that oxidative stress is one of the most important mechanisms mediating the developmental and behavioral toxicity of CPC to zebrafish larvae. Nevertheless, further study is still required to clarify the molecular mechanism and signaling pathway underlying the toxic effects of CPC on aquatic species in order to understand its ecological risks better.

## Figures and Tables

**Table 1:** Estimated coefficients for an accelerated failure time model describing the effects of cetylpyridinium chloride (CPC) on the time to death of zebrafish *(Danio rerio)* embryos within 120 h post-fertilization (hpf).

Factors	Coefficients (S.E.) ^1^	Wald-Z	Pr (>|Z|)
Intercept (*µ*)	5.73 (0.08)	69.3	<0.001
*d*_CPC_	−2.03 (0.01) × 10^−3^	−21.5	<0.001
Log (scale)	−0.64 (0.06)	−10.7	<0.001

^1^ The concentration of CPC was treated as a continuous variable when fitting the survival data; S.E.: standard error.

**Table 2:** Estimated coefficients for an accelerated failure time model describing the effects of cetylpyridinium chloride (CPC) on the time to hatching of zebrafish (*Danio rerio*) embryos.

Factors	CPC (Unit)	Coefficients (S.E.) ^1^	Wald-Z	Pr (>|Z|)
Intercept (*µ*)	0 µg/L	4.28 (0.003)	1371.5	<0.01
*d_CPC =_*_4.0_	4.0 µg/L	−0.5 (4.1) × 10^−3^	−0.13	0.900
*d_CPC =_*_40_	40 µg/L	1.5 (4.5) × 10^−3^	0.33	0.740
*d_CPC =_*_400_	400 µg/L	0.7 (4.3) × 10^−3^	0.17	0.860
Log (scale)		−3.85 (0.05)	−81.0	<0.01

^1^ The concentration of CPC was treated as a categorical variable when fitting the survival data; S.E.: standard error.

**Table 3:** Spearman’s correlation between the level of biomarkers for oxidative stress and the biological and behavioral parameters of zebrafish (*Danio rerio*) ^1^.

	Heart Rate		Average Velocity within Each 10 Min Period					Response to Light/Dark Shift			
	48 hpf	72 hpf	0–10 (D1)	10–20 (L1)	20–30 (D2)	30–40 (L3)	40–50 (D3)	L1/D1	D2/L1	L2/D2	D3/L2
ROS	−0.168	−0.825 **	−0.294	−0.133	−0.427	−0.350	−0.538	0.413	−0.839 **	0.727 **	−0.832 **
SOD	−0.224	−0.867 **	−0.252	−0.112	−0.259	−0.301	−0.252	0.364	−0.657 *	0.643 *	−0.643 *
GSH	−0.034	−0.741 **	−0.441	−0.273	−0.399	−0.385	−0.301	0.503	−0.524	0.587 *	−0.573
CAT	−0.034	−0.748 **	−0.168	−0.035	−0.329	−0.063	−0.469	0.238	−0.685 *	0.692 *	−0.776 **
MDA	0.011	−0.732 **	−0.235	−0.123	−0.389	−0.102	−0.518	0.277	−0.627 *	0.680 *	−0.732 **

^1^ Spearman’s correlation coefficients are listed, and the asterisks indicate statistical significance (* *p* < 0.05; ** *p* < 0.01). L: light period; D: dark period; ROS: reactive oxygen species; SOD: superoxide dismutase; GSH: glutathione; CAT: catalase; MDA: malondialdehyde.

## Data Availability

Data is contained within the article.
